# Rupture risk of intracranial aneurysms: Comparison between small ruptured intracranial aneurysms and large unruptured intracranial aneurysms

**DOI:** 10.1097/MD.0000000000038909

**Published:** 2024-07-12

**Authors:** Min-jie Peng, Lu Zeng, Lan-lan Liu, Li Wen, Guang-xian Wang

**Affiliations:** a Department of Pharmacy, Banan Hospital, Chongqing Medical University, Chongqing, China; b Department of Radiology, Banan Hospital, Chongqing Medical University, Chongqing, China; c Department of Radiology, Xinqiao Hospital, Third Military Medical University, Chongqing, China.

**Keywords:** computed tomography angiography, intracranial aneurysm, morphology, risk factor, rupture

## Abstract

To compare the differences in clinical and morphological features between small ruptured intracranial aneurysms and large unruptured intracranial aneurysms to evaluate the risk factors for the rupture of IAs. The clinical data of 189 consecutive patients with 193 IAs were reviewed. The patients and IAs were divided into ruptured (<5 mm) and unruptured groups (>10 mm). The characteristics of the patients and the intracranial aneurysms (IAs) were compared between the 2 groups, and the risk factors for rupture of IAs were assessed using multiple logistic regression. Patient age (odds ratio [OR], 0.955), IA located at the internal carotid artery (ICA, OR, 0.202), irregular shape (OR, 0.083) and parent vessel diameter (OR, 0.426) were negatively correlated with the risk of IA rupture. IAs located at bifurcations (OR, 6.766) were positively correlated with the risk of IA rupture. In addition to the size of the IAs, regardless of IAs shape, other factors, such as younger age (<63.5 years), location at a bifurcation, IAs located at the ICA and a small parent vessel diameter (<3.25 mm), can influence the risk of IA rupture.

## 1. Introduction

Intracranial aneurysms (IAs) are common, most of which are asymptomatic and never rupture during a patient’s lifetime.^[1]^ However, IA rupture is often associated with high mortality and high morbidity.^[2]^ Hence, accurate evaluation of rupture risk is crucial for informing the management of patients with unruptured intracranial aneurysms (UIAs). Traditionally, large IA often have a greater risk of rupture.^[3]^ A recent report also suggested that small UIAs (<5 mm) have low growth and low rupture rates and that very small UIAs (<3 mm) have little or no risk for rupture.^[4]^ Nevertheless, in clinical practice, small ruptured intracranial aneurysms (RIAs, ≤ 5 mm) account for 45% to 50% of total RIAs^[5]^; unexpectedly, many large UIAs (≥10 mm) remain stable for a long time. This means that IA size alone is not a reliable marker of rupture risk.

Assessment of IAs rupture risk is a complex matter and involvement a large number of factors. Some patient-specific clinical features, such as hypertension, age, a history of subarachnoid hemorrhage (SAH), presence of multiple IAs and smoking,^[5–8]^ and morphological features of IAs, such as shape and location,^[5–7,9,10]^ have been reported to be related to RIAs. However, other studies have found some different features. A study of the natural history of UIAs indicated that hypertension, a history of SAH, presence of multiple IAs and smoking did not significantly affect the risk of rupture.^[9]^ Surprisingly, the PHASES score (hypertension, age, earlier SAH, size, location, shape) considered earlier SAH to be a risk factor for IAs rupture,^[6]^ but the ELAPSS score (earlier SAH, location, age > 60 years, population, size, shape) found a negative correlation between earlier SAH and risk of growth of UIAs.^[10]^ In addition, there are significant differences between the 2 scoring systems for the specific site of the IAs.^[6,10]^

The size of the RIAs and UIAs was within the same range in previous studies, there may be interference with other factors. In addition to size, to better determine which clinical and morphological factors are associated with IAs rupture, we compared the differences in the clinical and morphological features between 2 extreme groups (RIAs, ≤5 mm vs UIAs, ≥10 mm).

## 2. Materials and methods

### 2.1. Subject selection

Our institutional ethics committee approved the present retrospective study and the requirement for informed consent from patients was waived. Patients with small RIAs (≤ 5 mm) and large UIAs (≥10 mm) were included. The exclusion criteria were as follows: mycotic, traumatic, or fusiform IAs; IAs that were associated with arteriovenous malformations or poor quality images; and patients with a UIA size smaller than 10 mm or an RIA size larger than 5 mm. Patients with UIAs were followed for at least 2 years via outpatient department visits or telephone surveys. For patients with multiple IAs, the RIA was confirmed based on nonenhanced CT, angiographic, or operative findings. Ultimately, a total of 189 consecutive patients (110 ruptured and 79 unruptured) with 193 IAs (110 ruptured and 83 unruptured) at 2 participating centers were retrospectively enrolled.

Clinical information (e.g. sex, age, smoking status, hypertension status) on all the patients was collected from electronic medical records and summarized for analysis by 2 assessors.

### 2.2. Imaging analysis

All computed tomography angiography (CTA) images were acquired on 64-slice CT machines (Revolution HD; GE Healthcare, Wisconsin; or LightSpeed VCT; GE Healthcare, Chicago). The CTA images were sliced to 0.625 mm, transferred to a GE Advantix workstation (Advantage Windows 4.5) to generate 3-dimensional volume renderings and then evaluated. All CTA images were evaluated separately by 2 observers (one with 10-year experience in vascular imaging and the other with 20 years of experience in neuroradiology), who blinded to patient information and stability status. Continuous data were calculated as average values. In cases of discrepancy in categorical data, discordance was resolved by a third reader (with 25 years of experience in neuroradiology).

**Figure 1.**
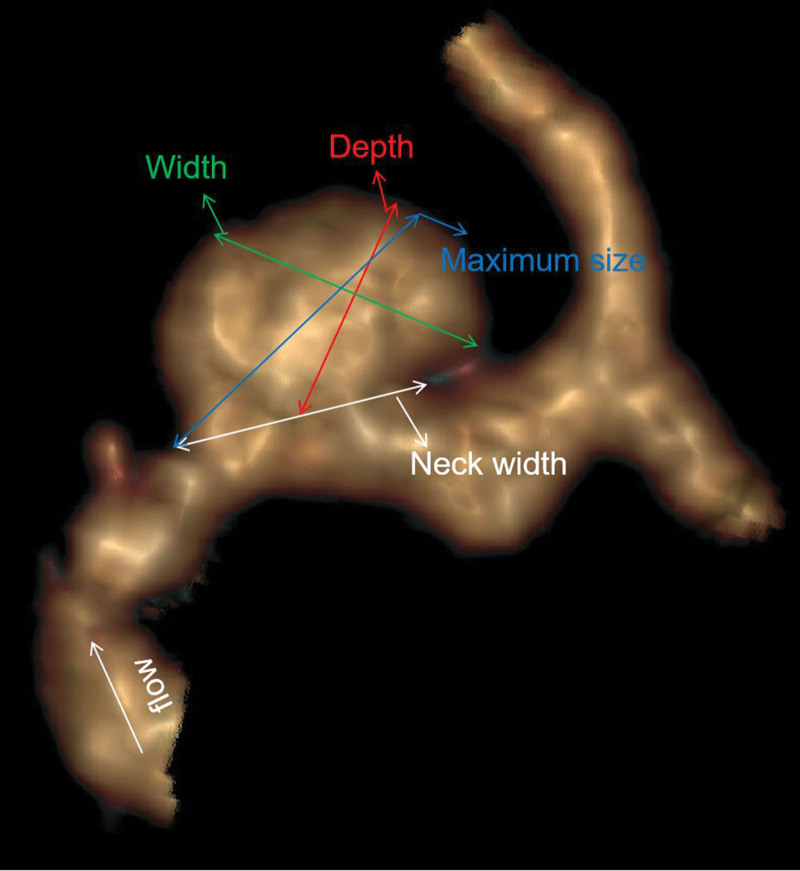
The image illustrates the method of dimension measurements: neck width (the largest cross-sectional diameter of the aneurysm neck); depth (the longest diameter between the neck and the dome); width (the maximum distance vertical to depth); and maximum size (the largest measurement in terms of maximum dome diameter or width).

Morphological parameters included IA location, the origin of the IA (sidewall or bifurcation type), shape (simple or irregular), size (maximum size, neck width, depth, and width), parent vessel diameter, aspect ratio (AR; depth/neck width), depth/width ratio (DW), size ratio (SR; depth/parent vessel diameter), and bottleneck factor (BF, width/neck width ratio). The IA location included the internal carotid artery (ICA), middle cerebral artery (MCA), anterior cerebral artery (ACA), anterior communicating artery (ACoA), posterior communicating artery (PCoA), and posterior circulation artery (PCA). The bifurcation type was defined as an IA located at major bifurcations in the cerebral vessel. An IA with an irregular shape was defined as an IA with a lobular or daughter sac. The maximum size was defined as the maximum diameter of an IA, the largest measurement in terms of maximum dome diameter or width. The neck width was defined as the width of the IA in the neck plane. The depth was defined as the longest diameter between the neck and dome. The width was defined as the maximum diameter vertical to the depth. The measurement of IAs is shown in Figure 1. These morphological parameters have been identified and described clearly in previous studies.^[7,11]^

### 2.3. Statistical analysis

SPSS 25.0 (SPSS Inc., Chicago) was used for the statistical analyses. Categorical data are presented as n (%) and were compared using Fisher exact tests. Continuous data are presented as the mean ± standard deviation and were compared using Mann–Whitney *U* tests or Student *t* tests, as appropriate. Multiple logistic regression analysis was used to assess the independent predictors of IA rupture. The cutoff values of the continuous data were calculated by the receiver operating characteristic curve with the maximum Youden index. A *P* value < .05 was regarded as statistically significant.

## 3. Results

The clinical data of the 189 patients are listed in Table 1. The mean age of the patients was 57.0 ± 13.0 years (54.3 ± 14.2 years for men and 58.4 ± 12.3 years for women). No significant differences were found between the 2 patient groups, except for a significantly younger age in the rupture group.

**Table 1:** The clinical data of patients with aneurysms.

Clinical data	Ruptured (n = 110)	Unruptured (n = 79)	*P*
Male	39 (35.5%)	23 (29.1%)	.433
Age (yr)	53.94 ± 11.36	61.33 ± 14.05	<.001
Hypertension	41 (37.3%)	27 (34.2%)	.759
Heart disease	5 (4.5%)	5 (6.3%)	.744
Diabetes mellitus	11 (10.0%)	7 (8.9%)	1.000
Cerebral atherosclerosis	1 (0.9%)	5 (6.3%)	.084
Current alcohol	27 (24.5%)	14 (17.7%)	.288
Current smoking	25 (22.7%)	19 (24.1%)	.863
Multiple aneurysms	11 (10.0%)	14 (17.7%)	0.133

Table 2 summarizes the morphological characteristics of the IAs. IAs located at the ACoA, ICA or bifurcation location and those with an irregular shape were associated with UIAs. Notably, almost all of the morphological characteristics except for the DW and parent vessel diameter in the ruptured group were smaller than those in the unruptured group.

**Table 2:** Morphologic characteristics of the aneurysms.

Morphologic characteristics	Ruptured (n = 110)	Unruptured (n = 83)	*P*
Location			
Internal carotid artery	7 (6.4%)	38 (45.8%)	<.001
Middle cerebral artery	18 (16.4%)	8 (9.6%)	.205
Anterior cerebral artery	8 (7.3%)	2 (2.4%)	.192
Anterior communicating artery	42 (38.2%)	9 (10.8%)	<.001
Posterior communicating artery	27 (24.5%)	19 (22.9%)	.865
Posterior cerebral circulation	8 (7.3%)	7 (8.4%)	.791
Bifurcation	88 (80.0%)	22 (26.5%)	<.001
Irregular shape	31 (28.2%)	61 (73.5%)	<.001
Maximum size (mm)	4.29 ± 0.66	17.17 ± 5.69	<.001
Neck width (mm)	3.17 ± 0.71	8.64 ± 3.11	<.001
Depth (mm)	3.54 ± 0.75	14.78 ± 5.21	<.001
Width (mm)	3.17 ± 0.73	14.39 ± 5.71	<.001
Parent vessel diameter	3.16 ± 0.77	4.03 ± 0.88	<.001
AR	1.18 ± 0.41	1.84 ± 0.70	<.001
DW	1.16 ± 0.29	1.09 ± 0.32	<.001
SR	1.37 ± 0.49	4.03 ± 1.56	<.001
BF	1.03 ± 0.28	1.78 ± 0.74	<.001

AR = aspect ratio, BF = bottleneck factor, DW = depth/width ratio, SR = size ratio.

Considering that the size of a UIA is significantly larger than that of an RIA, the parameters that are larger than that of an RIA were excluded. The factors that were significantly associated with the incidence of RIA were patient age, location at the ICA and ACoA, a bifurcation location, irregular shape, parent vessel diameter, and DW. Then, the 7 variables were entered into the forward stepwise multiple logistic regression model. Table 3 shows that the location at a bifurcation (odds ratio [OR] 6.766) strongly affected the risk of IA rupture, while patient age (OR 0.955), location at the ICA (OR 0.202), irregularity (OR 0.083) and parent vessel diameter (OR 0.426) were associated with a decreased risk of IA rupture.

**Table 3:** Multiple logistic regression model for prediction of small aneurysm rupture.

	OR	95% CI	*B*	*P*
Age	0.955	0.919–0.992	−0.046	.018
Internal carotid artery	0.202	0.052–0.786	−1.601	.021
Bifurcation	6.766	2.386–19.191	1.912	<.001
Parent vessel diameter	0.426	0.238–0.761	−0.853	.004
Irregular shape	0.083	0.031–0.219	−2.491	<.001
Anterior communicating artery	0.617	0.180–2.112	−0.482	.442
DW	1.759	0.431–7.172	0.564	.431

*B* = partial logistic coefficient, CI = confidence interval, DW = depth/width ratio, OR = odds ratio.

**Figure 2.**
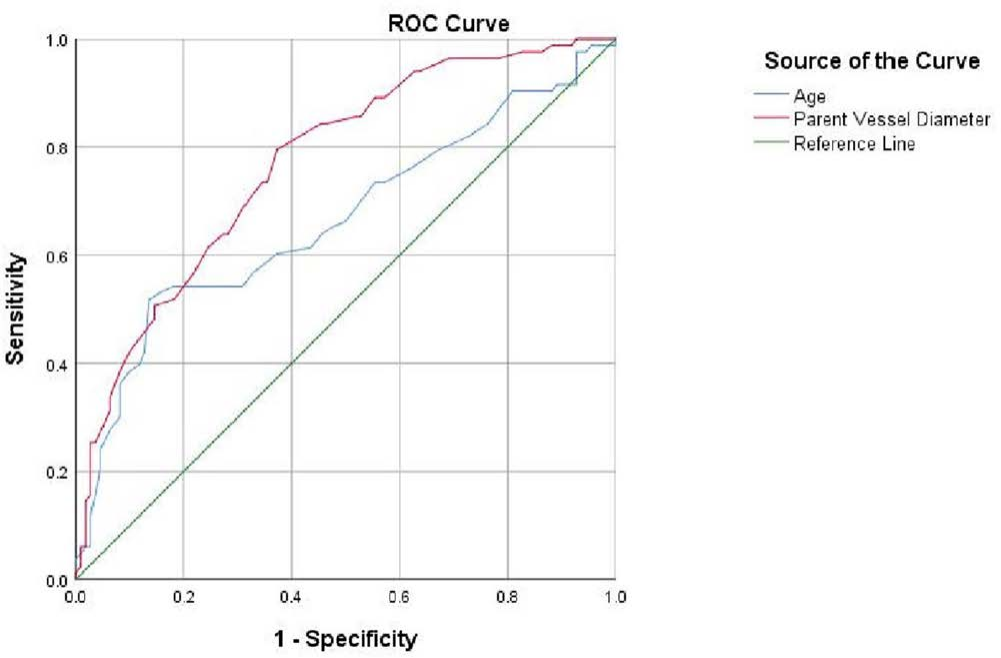
The areas under the receiver operating characteristic curves values for age and parent vessel diameter were 0.666 (95% confidence interval, 0.586–0.746) and 0.770 (95% confidence interval, 0.704–0.863), respectively. The cutoff points for age and parent vessel diameter were 63.5 years and 3.25 mm, the sensitivities were 54.2% and 79.5%, and the specificities were 87.2% and 62.7%, respectively. ROC = receiver operating characteristic.

The area under the receiver operating characteristic curve values for age and parent vessel diameter were 0.666 and 0.770, respectively, and the cutoff values were 63.5 years and 3.25 mm, respectively (Fig. 2).

## 4. Discussion

In this study, we compared small RIAs and large UIAs and found that regardless of IAs shape, patient age, location at the ICA, location in bifurcations, and parent vessel diameter were associated with a risk of IA rupture.

For many years, IA size has been considered the main factor associated with rupture risk and treatment, and small IAs are often left untreated and monitored by imaging. Many studies have suggested that small UIAs have low growth and rupture rates.^[3,4,7,11,12]^ However, according to recent reports, the actual number of small RIAs was considerable, and the percentages of total RIAs were up to 50%^[5]^ and 41%,^[13]^ respectively. This means that we cannot rely solely on the size of the IA to determine the rupture risk, while other clinical and morphologic characteristics should be noted. Therefore, we chose extreme contrast groups.

The role of patient age in the risk of IA rupture is unclear. Some studies have reported that patient age is not associated with IA rupture.^[4,9,11,12,14]^ Additionally, several studies have reported a positive correlation between age and IA rupture.^[6,15]^ However, recent studies have indicated that patient age is inversely related to IA rupture,^[5,7,8,16,17]^ which is consistent with this report. The inverse relationship between age and IA rupture may be associated with hemodynamics. With increasing age, the wall shear stress decreases because cerebral atherosclerotic or calcified walls slow the flow of blood into the IA.^[18]^ In this study, we found that patients aged <63.5 years were more prone to rupture. Unfortunately, there is not yet a specific age threshold for IA treatment.

IAs usually occur at the circle of Willis, especially at the ACoA, PCoA and ICA. Location is considered an important factor for IA treatment decisions.^[3]^ Moreover, an IA located at the ACoA and PCoA is believed to have a high risk of rupture.^[6,9,17,19]^ In this study, although the ACoA was found to be strongly associated with IA rupture in univariate analyses, this association was not significant according to multivariate regression analysis. One of the reasons may be that the small sample size limited the morphological analysis. Another reason may be associated with hemodynamics. Because an ACoA IA is also located at the bifurcation of the artery, the bifurcation type may be more important than the location. The rupture risk is different between sidewall-type and bifurcation-type IAs. Previous studies have reported that the bifurcated arterial wall is vulnerable because the wall is weak and because of hemodynamic stress changes in these regions.^[7,20]^ Moreover, IAs located at the ICA are less likely to rupture because they are usually the sidewall type.

In general, an IA with an irregular shape is more likely to become unstable or rupture because its irregular shape leads to unstable blood flow patterns.^[21]^ However, our present results show that an IA with an irregular shape has a lower risk of rupture. One of the reasons is that a large IA is more likely to lobulate, and a large diameter may counteract the instability of the blood flow pattern. Another reason is that small IAs are not yet lobulated or that shallow lobulation has been missed. This means that shape is not an important risk factor for small IAs rupture. However, the negative correlation between irregular shape and IA rupture is still debatable. This is most likely a statistical bias due to patient selection.

We found that a small parent vessel diameter (<3.25 mm) is associated with a greater risk of IA rupture. The reason may be that IAs arising from smaller vessels have thinner walls and may suffer greater wall tension.^[22]^ This also confirms that IAs located at the ICA are less likely to rupture because the ICA is often larger in diameter than other arteries.

This study had several limitations. First, we compared 2 groups of IAs. Patients with RIAs larger than 5 mm or UIAs smaller than 10 mm were excluded, which may have created biased in the selection of patients and may have led to statistical bias, and the results may not be applicable to other sizes of IAs. However, the aim of this study was to understand which factors, except for size, might contribute to IA rupture. Hence, the results may be more convincing. Second, the RIAs and UIAs had different statuses. The size and shape of the RIAs might be the result rather than the cause of IA rupture. Although no evidence has confirmed that IAs shrink after rupture, the results may be biased due to changes in size and shape after IA rupture. Third, some drugs, such as aspirin and statins, may reduce the incidence of IA rupture, and patient intake of such drugs has not been investigated.^[23]^ Fourth, this was a retrospective study with a small sample size, so there is a risk of bias. Fifth, our follow-up time needs to be extended, and some large UIAs may rupture in the future. Further prospective studies with larger patient samples could improve the significance of the findings.

In conclusion, we compared the clinical and morphological features between smaller RIAs and larger UIAs. We found that regardless of IAs shape, younger age (<63.5 years), location at a bifurcation, and a small parent vessel diameter (<3.25 mm) were independently associated with the rupture of IAs. IAs located at the ICA were less likely to rupture. These risk factors should be validated in future studies.

## Acknowledgments

This study was supported by the Joint Project of Science and Health of Chongqing City, China (2023MSXM022) and Banan District Science and Technology Bureau of Chongqing City, China (KY202208135759002).

## Author contributions

**Conceptualization:** Guang-xian Wang.

**Data curation:** Min-jie Peng, Lan-lan Liu.

**Funding acquisition:** Guang-xian Wang.

**Investigation:** Lu Zeng, Lan-lan Liu, Li Wen.

**Methodology:** Guang-xian Wang.

**Writing – original draft:** Min-jie Peng.

**Writing – review & editing:** Lu Zeng, Lan-lan Liu, Li Wen, Guang-xian Wang.
